# Cetylpyridinium chloride produces increased zeta-potential on *Salmonella* Typhimurium cells, a mechanism of the pathogen’s inactivation

**DOI:** 10.1038/s41538-019-0052-x

**Published:** 2019-10-16

**Authors:** Yagmur Yegin, Jun K. Oh, Mustafa Akbulut, Thomas Taylor

**Affiliations:** 1 Department of Nutrition and Food Science, Texas A&M University, College Station, TX 77843-2253 USA; 2 Artie McFerrin Department of Chemical Engineering, Texas A&M University, College Station, TX 77843-1136 USA; 3 Department of Animal Science, Texas A&M University, College Station, TX 77843-2471 USA; 4 Present Address: Department of Polymer Science and Engineering, Dankook University, 152, Jukjeon-ro, Suji-gu, Yongin-si, Gyeonggi-do 16890 Republic of Korea

**Keywords:** Applied microbiology, Agriculture

## Abstract

Cetylpyridinium chloride (CPC) is a quaternary ammonium sanitizer approved for fresh poultry animal carcass sanitization from microbial human pathogens, such as *Salmonella enterica*. Nonetheless, the interactions of CPC with *Salmonella* cells, and the mechanism of the sanitizer’s neutralization by lecithin remains largely unknown. This study aimed to investigate the interaction of CPC with lecithin and *Salmonella* Typhimurium to determine the interactions of the sanitizer and neutralizer impacting the bacterium’s survival. Application of 0.8% CPC is proposed to produce loss of microbial membrane integrity with loss of electrostatic repulsion between individual cells, resulting in the eventual emulsification of membrane lipids with cytoplasmic contents leakage. Our findings point to a two-phase interaction between CPC and lecithin impacting *S*. Typhimurium survival. The first consists of electrostatic attraction and charge neutralization between oppositely charged components of pathogen cell and CPC. The second involves formation of aggregates between sanitizer and pathogen, or between sanitizer, pathogen membrane lipids, and lecithin.

## Introduction

In the United States, the manufacture of fresh poultry products is regulated by the U.S. Department of Agriculture Food Safety and Inspection Service (USDA-FSIS). For poultry carcasses and fresh cut pieces, multiple chemical sanitizers are approved to decontaminate eviscerated carcasses and pieces from microbial foodborne pathogens, including *Salmonella enterica*.^1^ The quaternary ammonium sanitizer cetylpyridinium chloride (CPC) has been repeatedly studied and reported effective for the sanitization of poultry carcass and meat surfaces from microbial pathogens at up to 0.8%.^2–4^ Recent research has indicated that carryover of some sanitizers into poultry carcass sampling rinse fluids may prevent the successful detection of pathogenic microbes during routine verification testing.^5,6^ Consequently, recent changes to routine testing methods for poultry carcass testing to detect microbial pathogens have raised questions about the utility and necessity of chemical sanitizer neutralizing agents (i.e., neutralizers) and their impact on poultry processors’ ability to adhere to federal food safety performance standards for fresh poultry products.^7,8^ Dey and Engley^9^ previously incorporated lecithin into an antimicrobial neutralization formula for the purposes of counteracting QAC-type sanitizers. Mohammad et al.^10^ reported that the incorporation of soy lecithin at 7.0 g/L effectively neutralized CPC (0.8% w/v), facilitating *Salmonella* detection in a model microbiological medium.

The antimicrobial mechanisms of the sanitizer have been previously suggested to result from the insertion of alkyl chains into microbial membranes, resulting in membrane permeation and cytoplasmic leakage.^11,12^ Nonetheless, studies investigating the mechanisms of CPC antimicrobial activity against *Salmonella enterica* or other microbial pathogens on poultry carcass or meat surfaces are lacking in the scientific literature. Breen et al.^13^ reported CPC addition reduced or reversed *Salmonella* cell attachment to chicken skin samples, suggested to result at least partially from electrostatic interactions of the cationic surfactant with anionic headgroups and side groups on the bacterium’s outer membrane. Ma et al.^14^ using a CPC-fixing clay for testing antimicrobial activity of CPC against enterotoxigenic *E. coli* and *Salmonella* Typhimurium (ST), demonstrated cell morphology disruption by CPC application, as well as respiration inhibition in cells of both pathogens.

In addition to a general lack of data that describe mechanistic interactions of CPC with *Salmonella* or other human pathogenic bacteria, data are not known to be available detailing the interactions of the pathogenic microbe with the sanitizer CPC when a neutralizing agent such as lecithin is introduced. Understanding the interactions between these three agents would improve food safety specialists’ ability to accurately determine the reliability of poultry testing methods for pathogen detection. The objective of this research was to identify the key components of the mechanisms of CPC neutralization by lecithin to yield increased understanding of the interaction of sanitizer and neutralizer, as impacting ST survival. It was hypothesized by researchers that CPC would exert a surfactant-type antimicrobial activity, likely resulting in membrane permeabilization and/or lipopolysaccharide (LPS) release, and that lecithin would neutralize this by counter-acting or inhibiting CPC mixing within bacterial cell membranes.

## Results and discussion

### Size and ζ-potential of *S*. Typhimurium treated with CPC

**Fig. 1:**
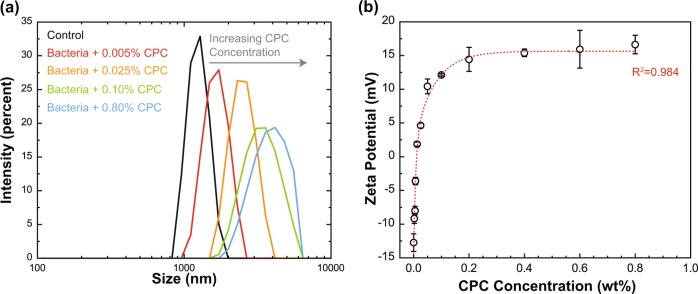
Hydrodynamic size (intensity averaged) of *Salmonella* Typhimurium as a function of concentration of CPC (**a**) obtained by dynamic light scattering and the change in the average ζ-potential (mV) of *Salmonella* Typhimurium cells with addition of CPC (**b**). Symbols depict mean values from three independent replications while error bars depict one sample s.d. (*N* = 3). The fitted trend line (dashed) is the two-phase (double) exponential decay model with the coefficient of determination of 0.984

**Fig. 2:**
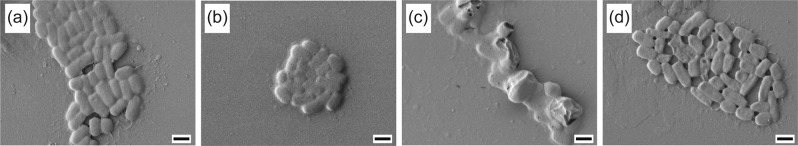
Scanning electron microscopy (SEM) images for 0.8% CPC-treated *Salmonella* Typhimurium cells treated by 0.8% CPC-treated cells at exposure times of 0 min (**a**), 1 min (**b**), 10 min (**c**), and 60 min (**d**). Images are representative of three independently completed experimental replications completed on differing days. Scale bar is 1 μm

Figure 1a depicts the impact of 0.005 to 0.8% CPC addition on the resulting sizes of *S*. Typhimurium cells in PBS, inoculated into reaction tubes at 9.1 ± 0.3 log_10_ CFU/ml. Untreated (control) ST cells displayed a relatively narrow distribution of size; the mean hydrodynamic radius of non-CPC-treated cells was 1.3 ± 0.07 µm in diameter (Fig. 1a). Addition of 0.8% CPC destabilized the outer membranes of *Salmonella* cells, likely the result of combined effects of charge neutralization and surfactant-based membrane lipid re-ordering. The addition of CPC broadened the size distribution of *Salmonella* cells, producing a wide unimodal distribution with a mean size of 4.0 ± 1.12 µm. This would indicate the sanitizer produced aggregation of cells, again likely due to charge neutralization and/or lipid re-ordering and release (Fig. 2). Lipids from *Salmonella* cell outer membranes were likely released via sanitizer application, resulting in large molecular aggregates of lipid and protein following cell death.

Likewise, mean ζ-potential of *Salmonella* cells not CPC-treated was electro-negative (–12.73 ± 1.31 mV) in PBS (Fig. 1b). After the bacterial cells were treated with different concentrations of CPC, ζ-potential immediately increased in double exponential fashion. The existence of such a trend is presumably due to the cationic amino groups of CPC and its covering of negatively charged surface components of cell membranes, as well as bacterial aggregation processes. There was a sharp increase observed in samples’ ζ-potential upon treatment with up to 0.2% CPC: a change from –12.73 ± 1.31 mV (0% CPC) to +14.43 ± 1.78 mV (0.2% CPC) (Fig. 1b). Above 0.2% CPC, the ζ-potential plateaued, asymptotically increasing to +16.63 ± 1.38 mV at 0.8% CPC. This indicates the full coverage or saturation of negatively charged functional groups on the cell outer surface. This could be due to the reduction of cell surface charge repulsion via covering over of anionic functional groups on the cell’s outer membrane, the colloidal stabilization of bacteria or the complexation and bridging of neighboring bacteria walls with oppositely charged CPC. Phosphates and carboxylic acid groups in lipopolysaccharides are responsible for the observed negative zeta potential.

### Lecithin addition impacts on CPC-treated *Salmonella*

**Fig. 3:**
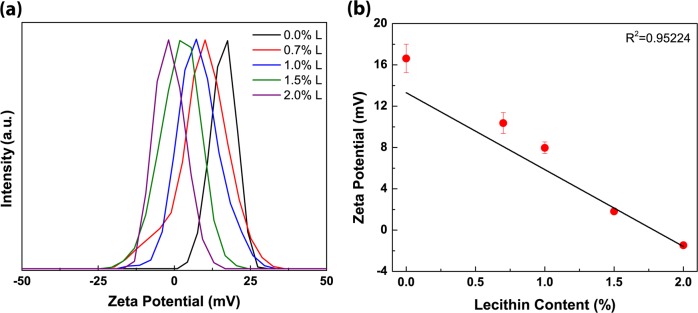
ζ-potential shifts depending on lecithin concentrations (**a**) and mean ζ-potential of *Salmonella* Typhimurium cells in the presence of lecithin after 0.8% CPC treatment. Values in panel (**b**) represent means of triplicate identical replications; error bars indicate one s.d. Fitted trend line depicts inverse relation of ζ-potential against increased lecithin addition, indicating increasing anionic characteristic of lecithin suspension

The influence of lecithin on the zeta-potential of 0.8% CPC-treated ST cells is shown in Fig. 3. Lecithin effect was measured at 0.7, 1.0, 1.5, and 2.0% lecithin to determine concentration dependency on observed effects. Lecithin was applied to 0.8% CPC-treated bacterial cells (1.0 min treatment period prior to neutralizer addition) and ζ-potential changes measured immediately thereafter. Addition of 1.5–2% lecithin reduced cationic charge distribution of samples, indicating the capacity of lecithin to neutralize CPC activity.

### CPC effect on *Salmonella* ζ-potential with 1.0% lecithin

**Fig. 4:**
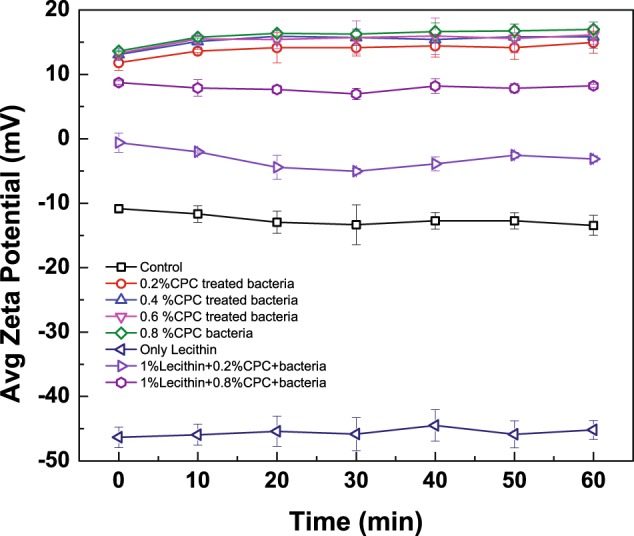
Change in ζ-potential of *Salmonella* Typhimurium cells immediately following mixing with CPC and 1.0% lecithin, over 60 min holding period at 25 °C. Symbols and connecting lines depict means of triplicate identical replications, while error bars depict one s.d. from sample means

Non-CPC-exposed ST ζ-potential readings were predictably electro-negative consistently throughout measurements (Fig. 4). Similarly, the neutralizer lecithin was also electro-negative, with a ζ-potential of approximately −45 mV. Samples of ST cells treated with increasing concentrations of CPC displayed increased ζ-potential, up to 11.8–13.6 mV, at sufficient concentrations overwhelming the surface charges of ST cells. Surface electrophoretic mobility (ζ-potential) of 0.2 or 0.8% CPC was significantly impacted by contact with up to 1.0% lecithin. The surface potential of a mixture of 0.2% CPC with 1.0% lecithin hovered around 0.0 mV, whereas 0.8% CPC with 1% lecithin ζ-potential ranged between 8.0 and 8.7 mV. ζ-potential values for sanitizer and lecithin mixtures increased as sanitizer concentration was increased from 0.2 to 0.8% (from 11.8 ± 1.2 mV at 0.2% CPC to 13.6 ± 0.1 mV at 0.8% CPC at 0 min incubation in sanitizer-treated cells). Mixing of CPC with lecithin effectively negated the anionic charges of lecithin. In comparison, when treated with CPC, the ζ-potential of cells treated with 1.0% lecithin and sanitizer increased in a similar fashion (from −0.6 ± 1.5 mV at 0.2% CPC in 1.0% lecithin-treated cells to 8.7 ± 0.2 mV at 0.8% CPC in 1.0% lecithin-treated cells) (Fig. 4). The increases in ζ-potential in both scenarios may indicate a mechanism of sanitizer activity, that of membrane surface charge disruption, in addition to permeabilization of the microbial membrane to water, ion, and leakage. Addition of lecithin following CPC application onto suspended cells, in reducing the ζ-potential, likely competed with the *Salmonella* cell membranes for interaction with CPC. CPC possessed strong ability to increase the surface charge of molecules and bacterial cells (Fig. 4), reducing ST ability to maintain proper respiration and metabolism.

### Survival of *Salmonella* treated with CPC and lecithin

**Fig. 5:**
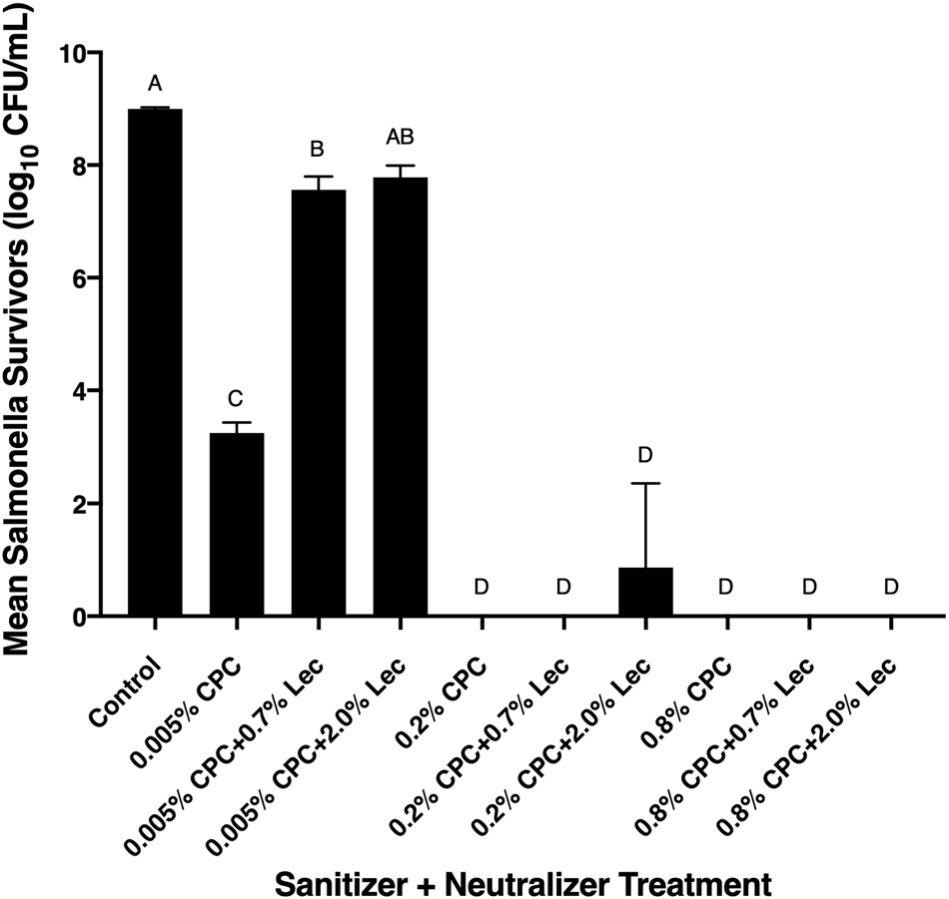
Least square means of *Salmonella* Typhimurium counts in presence of increasing CPC concentrations over a 1.0 min exposure period, with or without 0.7 or 2.0% Lecithin (Lec) exposure (40 min post lecithin incorporation exposure period). Bars depict means from triplicate identically completed replicates; error bars indicate one s.d. from means. Bars labeled with the same letter are not statistically different from each other (*p* < 0.05) by one-way analysis of variance and Tukey’s post hoc means separation test

The dependence of ST survival on CPC concentration, and the concentration of added lecithin, is presented in Fig. 5. The mean count of non-treated control bacteria was 8.99 ± 0.03 log_10_ CFU/mL, whereas no detection of ST survivors was achieved for 0.2 or 0.8% CPC treatments (limit of detection: 1 CFU/mL). Interestingly, these sanitizer concentrations produced electro-positive ζ-potential values for the bacteria/CPC systems. At 0.005% CPC treatment, the numbers of *S*. Typhimurium cells decreased from 8.99 to 3.24 log_10_ CFU/mL, a 5.76 log_10_ CFU/mL reduction. Also at 0.005% CPC, bacterial survival increased with addition of 0.7 or 2.0% lecithin, but not in a dose-dependent manner (Fig. 5). Thus, a small content of sanitizer in a liquid buffer led to a statistically significant decrease in the number of *Salmonella*, indicating a strong correlation between the number of bacteria and CPC treatment (*p* < 0.05). The lack of an apparent dose effect for 0.7 and 2.0% lecithin at the low concentration of sanitizer, however, indicates the neutralizer was sufficient to provide protection to ST cells, possibly by competing with ST cells for electrostatic interactions between anionic members of lecithin with the cationic surfactant, or by formation of structures wherein lecithin sequestered CPC from ST cells. At higher concentrations of sanitizer (0.2 and 0.8%), however, even 2.0% lecithin was generally unable to overcome the inactivation of the microorganism by the sanitizer. Even though lecithin was added at 1 min after addition of CPC to ST cells, inactivation of the pathogen occurred quickly, also suggested in Fig. 2. The general lack of pathogen survival at higher CPC doses, even when lecithin was added at higher concentrations, suggests that if a dose effect is to be observed, it will be at a lecithin concentration substantially higher than that approved by the USDA-FSIS in its nBPW formulation (0.7% w/v). Additionally, it may require lecithin to contact *Salmonella* cells prior to CPC, unlikely to occur given the sequence of sanitizer and neutralizer use in commercial poultry harvest and routine testing.

Hamouda and Baker, Jr.^15^ investigated the antimicrobial mechanism of action of 8N8, a negatively charged water-in-oil emulsion, and W60C, a cationic liposome, against the Gram-negative bacteria *Escherichia coli* and *Vibrio cholerae*. Their study demonstrated the positively charged W60C showed much stronger antimicrobial activity than the anionic 8N8 against negatively charged Gram-negative bacteria when divalent cations were chelated. In the current study, we utilized distilled deionized water, reducing the potential for cations to inhibit the attraction of CPC to *S*. Typhimurium cell surfaces. Interactions between bacteria and cationic and anionic surfactants were also investigated by Zhang et al.^16^ The cationic surfactant, tetraphenylethene-dodecyltrimethylammonium bromide (TPE-DTAB), showed high interaction with *Escherichia coli* by fluorescence microscopy, while the anionic surfactant tetraphenylethene-sodium dodecyl sulfonate (TPE-SDS) did not show any interaction. An electro-positive surface ζ-potential of TPE-DTAB (when in excess versus TPE-SDS) likely resulted in electrostatic attraction to *E. coli* cells membrane surfaces, followed by long alkyl chain of the surfactant inserting into bacterial membrane and producing leakage of cytoplasmic contents.^16^ However, negatively charged TPE-SDS did not attract bacteria and could not come closer to the negatively charged bacteria due to electrostatic repulsion between cell surface and surfactant.

It is important to highlight that at very low concentration (0.005%; 0.00015 M)), CPC (exposure period of 1.0 min) was able to produce a 5.76 log_10_ CFU/mL reduction in ST cells in the absence of lecithin, indicating strong potency of CPC as a sanitizer, despite being slightly above the critical micelle concentration (CMC) in water (0.00012 M).^17^ Antimicrobial efficacy of sanitizers is impacted by organic load encountered during poultry processing, such as fat and protein content in poultry immersion-type chilling waters. Organic loads in immersion chilling tanks can decrease the efficiency of sanitizers, potentially requiring elevated sanitizer concentrations to overcome inactivation by organic matter. In the current study, aggregation of membrane components of *Salmonella* was observed when surface charge of suspended bacteria was turned to electro-positive due to the addition of excess CPC, a cationic surfactant.

### Impact of CPC and lecithin on cell appearance and morphology

**Fig. 6:**
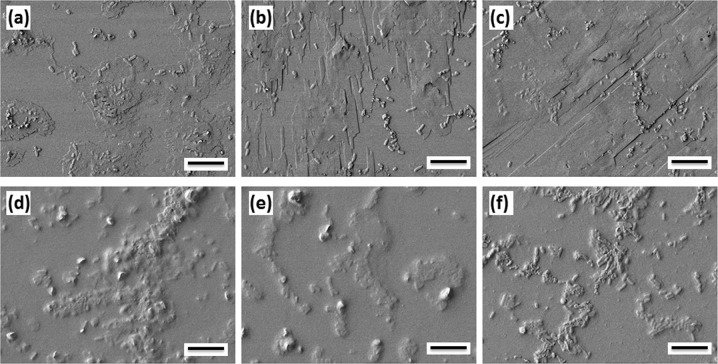
Scanning electron microscopy (SEM) images (**a**–**c**) for 0.8% CPC-treated *Salmonella* Typhimurium cells and (**d**–**f**) lecithin effect for 0.8% CPC-treated cells for different exposure times for 1 min (**a**, **d**), 10 min (**b**, **e**), and 60 min (**c**, **f**). Images are representative of three independently completed experimental replications completed on differing days. Scale bar is 10 μm

In experiments determining the impact of sanitizer with subsequent neutralizer addition to ST cells on cellular shape and morphology changes, micrograph images were collected at 1, 10, and 60 min following treatment with sanitizer (Fig. 2, Fig. 6a–c). For ST cells treated only by 0.8% CPC, as the exposure time was increased, sanitizer-treated cells appeared to initially aggregate (Fig. 2b) and membrane lipids emulsify (Fig. 2c), potentially due to surface charges being covered by the cationic surfactant. *Salmonella* cells lost cell structure during prolonged exposure to the sanitizer (Fig. 2c, d; Fig. 6a–c). Conversely, Fig. 6 panes d–f show embedded bacterial cells and cell matter within a layer of lecithin (added after 1.0 min CPC application at 0.8%). SEM images were quite different after lecithin addition as compared to only CPC-treated bacteria. Application of lecithin into the sample vessel resulted in a layer of surfactant forming on the glass slide, covering the remaining cells, and likely furthering emulsification of the membrane lipid components of ST cells. On solid surfaces, addition of lecithin could provide protection to ST cells by covering susceptible membrane components prior to sanitizer insertion, should the neutralizer contact the microbial cell prior to sanitizer contact. This would potentially give rise to increased pathogen survival, as reported in other research detailing neutralization of CPC by lecithin.^6^ In free-swimming cells, such as those which might be found in poultry carcass rinse fluids, addition of lecithin might be expected to form complexes with CPC rather than forming a protective coating on surface-adhered bacteria, given sufficient content of lecithin.^5^

CPC has been reported effective for reducing the numbers of *Salmonella enterica* or other bacterial pathogens on surfaces of poultry carcass or cut pieces.^2,18^ Gerba^19^, citing McDonnell^20^, described early steps in quaternary sanitizer antimicrobial mechanisms against bacteria, indicating requirements for attachment and penetration of the outer membrane in Gram-negative bacteria and/or the cytoplasmic membrane in Gram-negative and -positive bacteria, following membrane lipid emulsification and disorganization. However, impacts of the cationic charge component on bacterial surface charge were not discussed. Similarly, other researchers have more recently indicated biscationic QACs demonstrated greater antimicrobial activity against the human enteric pathogen *Campylobacter* versus monocationic QACs (including CPC). The increased charge of the bis-cationic QACs led to greater pathogen reduction within the experimental period compared to CPC and other monocationic QACs.^21^ On the other hand, research into the influence of CPC treatment on bacterial adherence to oil/water interfaces with the bacterium *Pseudomonas fluorescens* reported little change in cell membrane hydrophobicity following CPC treatment (200 mg/L), though cell surface ζ-potential was significantly changed, similar to findings in the current study.^22^ These researchers suggested that influences of the cationic sanitizer on cell surface ζ-potential, specifically charge neutralization through interactions of oppositely charged components, likely led to increased adhesion and interaction with the oil/water interface. A similar impact was observed here, as changes in cell surface charge of CPC-treated *Salmonella* were observed, and loss of surface charge led to increased observed membrane lipid components reorganizing and/or aggregating together.

Quisno et al.^23^ reported the inclusion of lecithin as effective for the neutralization of bacteriostatic activity of cationic surfactants and other cationic disinfectants, though no mechanism of activity was suggested. Recent research indicates the inclusion of sanitizer neutralizers, such as lecithin, for the neutralization of quaternary ammonium sanitizers like CPC, increases the likelihood of *Salmonella* recovery during poultry carcass or parts testing.^5,24^ The formation of lecithin/CPC mixed micelles was not detected in the current study by DLS or ζ-potential analysis when mixed with ST cells, though mixing of the surfactants potentially occurred, given changes in ζ-potential for lecithin:CPC mixtures where 1% lecithin was mixed with CPC at differing concentrations. Microscopy indicated mixing of sanitizer along with bacterial membrane components, likely leading to cell death through cytoplasmic contents leakage via membrane integrity loss.

In the current study, we present hydrodynamic radius, ζ-potential (electrophoretic mobility), microbiological and microscopic data describing the interactions of the cationic poultry sanitizer CPC with the bacterium ST, with or without the inclusion of the sanitizer neutralizer lecithin. Bacterial cells were reduced to non-detectable counts (from a starting load of 8.99 ± 0.03 log_10_ CFU/ml with a contact time of 1 min) at concentrations of CPC of 0.2% (2000 ppm), but demonstrated sanitizer concentration-dependent survival at very low concentration to the sanitizer (0.005% CPC). Addition of CPC resulted in increased ST ζ-potential, likely a result of outer membrane component negative charges being covered by the cationic quaternary amino group in the CPC. These data indicate CPC produces inhibition of the pathogen through initial charge attraction to electro-negative components on the *Salmonella* surface, and at sufficient concentration, charge neutralization leads to loss of membrane component ordering and organization. The capacity of alkyl chain components on CPC to insert into bacterial membranes was not directly investigated in this study, but possibly added to the observed antimicrobial activity of the sanitizer. Addition of lecithin at up to 2% potentially provided some degree of neutralization to CPC by competing for charge attraction with *Salmonella* cells, though plate count data indicate even this concentration of neutralizer was insufficient to afford pathogen survival post-CPC exposure. The use of sanitizer neutralizers during poultry carcass and cut pieces routine sampling has been reported necessary to improve the accuracy of testing for *Salmonella* and *Campylobacter* by the USDA-FSIS^8^. In the current study we propose a mechanism of CPC interaction with *Salmonella*, that being the change in surface charge of treated cells via CPC covered anionic components of the cellular cytoplasmic membrane, resulting in disorganization and membrane integrity loss. Lecithin as a neutralizer, at lower CPC concentrations, competed for electrostatic attraction with CPC and *Salmonella* membrane components, though at higher concentrations of CPC, lecithin at concentrations used in USDA-FSIS routine testing media was insufficient to neutralize all sanitizer activity via charge neutralization or mixed micelle complex formation, determined by *Salmonella* inactivation by CPC.

## Methods

### Bacterial isolate preparation

*Salmonella enterica* serovar Typhimurium (ST) Leeligen Type (LT) 2 was revived from cryo-storage (−80 °C) from the culture collection in the Food Microbiology Laboratory, Department of Animal Science, Texas A&M AgriLife Research (College Station, TX, USA) by aseptically inoculating a loop of preserved culture into 10.0 mL steam-sterilized (121 °C, 15 min) tryptic soy broth (TSB; Becton, Dickinson and Co., Sparks, MD, USA), and incubating statically for 24 h at 35 °C. This isolate was chosen to accommodate Texas A&M University Institutional Biosafety Committee requirements for biosafety level (BSL) 1 containment within microscopy and physico-chemical analytical laboratories within the Artie McFerrin Department of Chemical Engineering, Texas Engineering Experiment Station (College Station, TX, USA). Following 24 h of incubation, a loopful (10.0 μL) of overnight culture was aseptically sub-cultured in 10.0 mL of sterile TSB and incubated in similar fashion for 24 h at 35 °C.

### Preparation of sanitizer and neutralizer reagents

Cetylpyridinium chloride (CPC; Cecure®: 40% active agent per manufacturer guidance) was provided by Safe Foods Corp., N. Little Rock, AR, USA); it was diluted in sterile distilled, deionized water to produce suspensions of 0.2, 0.4, 0.6, and 0.8% CPC for experimentation. Additionally, sterile distilled, deionized water was utilized in order to prepare 0.0% CPC control samples. Working solutions of the sanitizer were prepared to deliver increasing concentrations of sanitizer; CPC was diluted to produce the concentrations of CPC (0.002, 0.005, 0.006, 0.013, 0.025, 0.05, 0.1, 0.2, 0.4, 0.6, and 0.8%) upon addition to reaction tubes containing bacterial cells. Refined soy lecithin (reagent grade) was purchased from Alfa Aesar (Ward Hill, MA, USA), and was prepared in sterile distilled, deionized water in order to deliver up to 1.0% lecithin upon mixing with CPC-containing samples, with or without ST cell addition. Lecithin maximal content was chosen based on USDA-FSIS incorporation of 7.0 g/L (0.7% w/v) lecithin in the formula of neutralizing buffered peptone water (nBPW) for the rinsing of poultry carcasses and fresh cut pieces.^25^

### Light scattering analysis of ST treated by 0.005 to 0.8% CPC

Revived ST cells were centrifuged at 4000 rpm on a bench-top mini-centrifuge for 15 min at ambient condition (25 °C) to produce a bacterial pellet. Following centrifugation, the supernatant was poured off and cell pellets were suspended in one volume of sterile phosphate-buffered saline (PBS; Thermo-Fisher Scientific, Waltham, MA, USA). Three identically completed centrifugation and washing procedures were completed, after which cells were serially diluted in PBS and enumerated on 3M™ Petrifilm™ Aerobic Count Plate films to verify the number of ST cells in the reaction tube were ~9.0 log_10_ CFU/mL. Inoculated films were incubated 24 h at 36 ± 1 °C prior to colony counting. Following enumeration of cells, reaction tubes containing ST cells were mixed with CPC-containing solution prepared to deliver 0.005, 0.025, 0.1, or 0.8% sanitizer upon addition to the culture-containing tube, with a 1 min exposure period to the sanitizer. Immediately thereafter, cells were loaded into a ZS90 Zetasizer Instrument (Malvern Instruments, Ltd., Westborough, MA, USA) for dynamic light scattering (DLS) analysis of cell size. The measurements were carried out at a scattering angle of 90° at 25 °C.

### ST surface ζ-potential change by CPC and lecithin exposure

Following initial DLS analysis of ST cells with and without treatment by 0.8% CPC, analysis was made of the impact of systematically increasing concentrations of CPC (0.0, 0.002, 0.005, 0.006, 0.013, 0.025, 0.05, 0.1, 0.2, 0.4, 0.6, and 0.8%) on outer surface ζ-potential (electrophoretic mobility) of ST cells. Cell surface ζ-potential was tracked at multiple time increments over a 60 min period at ambient temperature using a Zeta-Sizer ZS90 Instrument (Malvern Instruments, Ltd.) after bacterial cells were treated with CPC. ST ζ-potential measurements were performed in 0.5 mM PBS (pH 7.31 ± 0.02) to minimize the impact of pH fluctuations. The impact of lecithin inclusion was measured at 2.0, 1.5, 1.0, and 0.7% lecithin to determine concentration dependency on observed ζ-potential. Lecithin was applied to 0.8% CPC-treated bacterial cells (1.0 min treatment period prior to neutralizer addition) and ζ-potential changes measured immediately thereafter. ζ-potential measurements were collected continuously until stable.

### Enumeration of ST cells treated by CPC and lecithin

ST cells were prepared as described above. Differing concentrations of lecithin (2.0, 0.7, and 0.0%) were applied to ST cells pre-exposed for 1.0 min at ambient temperature condition (25°) to 0.005, 0.2 or 0.8% CPC (CPC content at which ST cells ζ-potential became constant, intermediate CPC concentration, and maximum allowable CPC concentration allowed for poultry sanitizing, respectively). Following lecithin addition, a 40.0 min holding period was completed prior to enumeration of surviving bacterial cells. Surviving ST cells were enumerated on tryptic soy agar (TSA; Becton, Dickinson and Co.) following preparation of serial dilutions in phosphate-buffered saline (PBS; Thermo-Fisher Scientific, Waltham, MA, USA) and incubating aerobically for at least 24 h at 37 °C. Resulting plate counts were log_10_-transformed for purposes of statistical analysis.

### Visualization of CPC and lecithin-treated ST cell morphology

Microscopic images were obtained by scanning electron microscopy (SEM) using a JSM-7500F electron microscope (JEOL, Tokyo, Japan), in order to visualize any changes in ST cell shape and morphology as a function of sanitizer and lecithin application. The samples were coated with 15 nm platinum/palladium (Pt/Pd) to eliminate any positive charging effects. The SEM was operated at an accelerating voltage of 1.0 kV and emission current of 20 μA. SEM images were taken of *Salmonella* cells after 1, 10, and 60 min treatment with 0.8% CPC. After the treatment cells were thoroughly rinsed in sterile milli-Q water to remove CPC residue. Identically prepared ST cells were then subjected to 0.8% CPC treatment (1.0 min) and then treated with 0.7% lecithin, after which micrographs were collected after 1, 10, and 60 min of lecithin exposure.

### Data analysis

All DLS, ζ-potential, and plating (cell enumeration) experiments were replicated three times in identical fashion over differing days (*N* *=* 3). Additionally, SEM imaging was completed for three identically prepared independent sets of samples over three differing dates. Statistical analysis of data was completed using ORIGIN® v.8 software (OriginLab Corp., Northampton, MA, USA). All microbiological data were log_10_-transformed prior to statistical analysis. One-way analysis of variance (ANOVA) with Tukey’s Honestly Significant Differences (HSD) post hoc test was used to determine significant differences in data between the treatments at a significance level of *P* < 0.05.

### Reporting summary

Further information on research design is available in the Nature Research Reporting Summary linked to this article.

## Supplementary information

Reporting Summary

## Data Availability

Data will be made available by request to co-senior authors.

## Supplementary information

**Supplementary information** accompanies the paper on the *npj Science of Food* website (10.1038/s41538-019-0052-x).
